# Association between Baltic sea diet and healthy Nordic diet index with risk of type 2 diabetes mellitus: a case–control study

**DOI:** 10.3389/fendo.2025.1510427

**Published:** 2025-03-27

**Authors:** Amr Ali Mohamed Abdelgawwad El-Sehrawy, Mahmood Jawad, Yasir Mohammed Hammood, Suhas Ballal, Manish Srivastava, Jaafaru Sani Mohammed, Renu Arya, Rishiv Kalia, Jawad Kadhim Ahmed, Muthena Kareem

**Affiliations:** ^1^ Diabetes Endocrinology and Metabolism, Internal Medicine, Diabetes, Endocrinology and Metabolism University, Mansoura University, Mansoura, Egypt; ^2^ Department of Pharmacy, Al-Zahrawi University College, Karbala, Iraq; ^3^ College of Physical Education and Sport Science, Almaarif University, Anbar, Iraq; ^4^ Department of Chemistry and Biochemistry, School of Sciences, Jain University, Bangalore, Karnataka, India; ^5^ Department of Endocrinology, National Institute of Medical Sciences, NIMS University Rajasthan, Jaipur, India; ^6^ Medical Analysis Department, Faculty of Applied Science, Tishk International University, Erbil, Iraq; ^7^ Chandigarh Pharmacy College, Chandigarh Group of Colleges-Jhanjeri, Mohali, Punjab, India; ^8^ Centre for Research Impact and Outcome, Chitkara University Institute of Engineering and Technology, Chitkara University, Rajpura, Punjab, India; ^9^ Department of Medical Laboratories Technology, AL-Nisour University College, Baghdad, Iraq; ^10^ Department of Medical Analysis, Medical Laboratory Technique College, The Islamic University, Najaf, Iraq; ^11^ Department of Medical Analysis, Medical Laboratory Technique College, the Islamic University of Al Diwaniyah, Al Diwaniyah, Iraq; ^12^ Department of Medical Analysis, Medical Laboratory Technique College, the Islamic University of Babylon, Babylon, Iraq

**Keywords:** Baltic Sea diet, healthy Nordic diet index, type 2 diabetes mellitus, T2DM, dietary indices

## Abstract

**Backgrounds:**

Recent evidence shows the beneficial effects of Baltic Sea diet score (BSDS) and healthy Nordic diet index (HNDI) on chronic diseases; however, there is no evidence to investigate them on the risk of type 2 diabetes mellitus (T2DM). The purpose of this study was to investigate the associations between BSDS and HNDI with the risk of T2DM.

**Methods:**

This study used a case-control design with participants aged 18 to 60 diagnosed with type 2 diabetes in the last six months (225 cases, 450 controls). The evaluation of BSDS and HNDI employed a validated 168−item semi−quantitative food frequency questionnaire (FFQ). Binary logistic regression was used to determine how OBS and T2DM are related.

**Results:**

The mean scores for the BSDS and HNDI were 16.00 ± 2.49 and 11.99 ± 2.61, respectively. The final model, which accounted for confounding variables, indicated that increased adherence to the HNDI is associated with a reduced likelihood of developing T2DM (OR = 0.42; 95% CI 0.18–0.98; p for trend = 0.043). Additionally, a significant association was observed between lower likelihood of T2DM and higher BSDS scores in both unadjusted (OR = 0.49, 95% CI 0.31–0.77; p for trend = 0.001) and adjusted (OR = 0.48, 95% CI 0.32–0.89; p for trend = 0.003) models.

**Conclusion:**

Our research shows that following a Nordic diet significantly reduces the risk of T2DM. Commitment to this dietary pattern may also reduce related risk factors. Further longitudinal studies across diverse populations are needed to validate these findings.

## 1 Introduction

The rising prevalence of type 2 diabetes (T2D) and its association with various modifiable lifestyle factors, particularly nutrition, underscores its significance as a global health issue (1). Worldwide, millions of individuals are affected by type 2 diabetes, and as dietary practices and lifestyles evolve, incidence rates are also affected (2). A comprehensive understanding of the dietary determinants of type 2 diabetes is essential for the development of effective preventive interventions. This research specifically examines two dietary patterns: the Baltic Sea Diet and the Healthy Nordic Food Index, with the aim of assessing their potential benefits in reducing the risk of type 2 diabetes.

Recent statistics indicate that type 2 diabetes (T2D) affects approximately 422 million individuals globally, a figure projected to rise due to increasing obesity rates, aging populations, and sedentary lifestyles (3). The global burden of T2D underscores the urgent necessity for effective preventive strategies, particularly through modifiable lifestyle factors such as nutrition (4). Nutritional choices play a pivotal role in both the onset and management of T2D. The consumption of foods high in refined carbohydrates and saturated fats, coupled with low fiber intake, has been linked to an elevated risk of developing diabetes. Conversely, research indicates that a dietary pattern characterized by an abundance of fruits, vegetables, whole grains, and lean proteins significantly reduces this risk (5, 6).

The Baltic Sea Diet closely aligns with dietary recommendations for the prevention of type 2 diabetes, emphasizing the consumption of whole grains, seafood, fruits, and vegetables (7). A Nordic study demonstrated a negative correlation between adherence to this diet and the incidence of type 2 diabetes (8). Furthermore, the health effects of items included in the Healthy Nordic Food Index, commonly consumed in the Nordic region, have been evaluated (9). Research indicates that reduced inflammation and improved glycemic control may underlie the association between lower scores on this index and a diminished risk of type 2 diabetes (10).

Comparative research indicates that the risk of developing type 2 diabetes can be significantly reduced through adherence to both the Healthy Nordic Food Index and the Baltic Sea Diet. A critical analysis reveals that individuals who followed these dietary patterns exhibited substantially lower incidences of type 2 diabetes compared to those who did not engage in these dietary practices (11, 12).

This study aims to enhance nutritional guidelines and public health policy by examining how specific dietary patterns affect type 2 diabetes risk. Promoting the Baltic Sea Diet and Healthy Nordic Food Index can help healthcare professionals guide communities toward healthier eating, preventing T2D and improving metabolic health.

## 2 Methods

### 2.1 Study population

This study utilized a case-control design, targeting participants aged 18 to 60 years who had been diagnosed with type 2 diabetes within the prior six months (225 cases and 450 controls). The diagnosis was established according to recognized criteria pertaining to glucose levels, specifically including fasting blood sugar (FBS) levels of 126 mg/dl or higher and 2-hour post-glucose (2 h-PG) levels of 200 mg/dl or greater, as indicated in previous research (13). The study included not only diabetic participants but also healthy individuals within the same age range, who were required to meet specific glucose level criteria. For the healthy participants, fasting blood sugar (FBS) levels were mandated to be below 100 mg/dl, and 2-hour post-glucose (2 h-PG) levels had to remain under 200 mg/dl, consistent with prior research (13). To preserve the integrity of the findings, several exclusion criteria were implemented. Individuals with certain chronic diseases, as well as those diagnosed with Type 1 diabetes or gestational diabetes, were excluded. Furthermore, participants adhering to specific dietary regimens or taking particular medications were also omitted from the study. Pregnant and breastfeeding women were deemed ineligible for participation in the study. Additionally, individuals with a familial history of diabetes or hypertension were excluded. Furthermore, participants who did not complete the food frequency questionnaire, which comprised over 35 items, or whose reported energy intake fell outside the designated range of 800 to 4200 kilocalories were also excluded from the analysis.

### 2.2 Dietary assessment

Dietary intake data was collected through personal interviews and a semi-quantitative food frequency questionnaire (FFQ) with 168 food items, assessing consumption over the past year. For participants in the case group, this timeframe specifically referred to the year leading up to their diagnosis, while for individuals in the control group, it pertained to the year preceding their interview. A nutritionist assisted in converting food sizes to grams, and total intake was standardized to a daily rate. Nutrient composition was analyzed using modified Nutritionist IV software for comprehensive dietary assessment.

### 2.3 Dietary indices

Developed by Kanerva et al. (14), the Baltic Sea Diet Score (BSDS) comprises nine components: 1) fruits and berries; 2) vegetables (roots, pulses, etc.); 3) cereals (excluding rice and pasta); 4) fish; 5) processed and unprocessed meats; 6) low-fat or fat-free milk; 7) the ratio of PUFAs to SFAs and trans fatty acids; 8) alcohol (excluded); and 9) total fat as a percentage of total energy intake. Each component was categorized into three tertiles (Q1, Q2, Q3), with healthy items scored 1-3 (low to high adherence) and harmful items (meat and total fat) scored inversely. A higher BSDS score (0-24 points) represents greater overall adherence to the Baltic Sea Diet.

The initial assessment of the Healthy Nordic Diet Index (HNDI) was carried out by Olsen et al. (15), who utilized a specific collection of six distinct items for their analysis. These items included fish, cabbage, a variety of vegetables, whole grains, oats, as well as apples, pears, fruits that possess high antioxidant properties, and root vegetables. Within the framework of this index, the six components are systematically organized into tertiles, which are designated as Q1, Q2, and Q3, based on the levels of intake observed among individuals. Following this categorization, the tertiles, which range from Q1 to Q3, are allocated scores of 1, 2, and 3, respectively, reflecting the degree of consumption. The adherence level to the Healthy Nordic Diet Index is quantified on a scale that spans from 0 to 18, where higher numerical values of the HNDI correspond to a greater degree of adherence to the dietary guidelines established.

### 2.4 Anthropometric measurement

Using a SECA 700 Digital Scale (rounded to the nearest 100 grams), weight measurements were taken with participants wearing minimal clothing and removing shoes. Height was measured using a Seca portable gauge (accurate to 0.1 cm). Waist circumference was assessed from the iliac crest to the last rib, and hip circumference at maximum buttock fullness. BMI was then calculated by dividing weight (kg) by height (m) squared.

### 2.5 Statistical analysis

Statistical analyses, using SPSS version 21, assessed the association between Healthy Nutritional Dietary Index (HNDI) and Body Shape Distress Scale (BSDS) scores and Type 2 Diabetes Mellitus (T2DM) risk. Data normality was checked using the Kolmogorov-Smirnov test and histogram analysis. Baseline characteristics and dietary intakes (means and standard deviations for quantitative variables; counts and percentages for qualitative variables) were analyzed using independent sample t-tests and chi-squared tests. Logistic regression analysis explored the HNDI and BSDS-T2DM association, adjusting for confounding factors (gender, BMI, waist circumference, hip circumference, physical activity, smoking status, education, drug use, illness history, caloric intake, and dietary fiber). Odds ratios (ORs) with 95% confidence intervals (CIs) were calculated across quartiles, with p<0.05 indicating statistical significance.

## 3 Results

**Table 1** presents the participants’ basic characteristics and biochemical data, organized by BSDS and HNDI quartiles. The study population’s average age was 39.53 ± 9.79 years, and average BMI was 27.10 ± 4.45 kg/m². Mean BSDS and HNDI scores were 16.00 ± 2.49 and 11.99 ± 2.61, respectively. Individuals in the highest quartiles of BSDS and HNDI were significantly older and showed marked differences in educational attainment compared to those in the lowest quartiles. No other statistically significant differences were observed between the quartiles of these indices and other factors.

**Table 1:** The demographic characteristics across the quartiles of the Healthy Nordic Diet Index (HNDI) and the Baltic Sea Dietary Score (BSDS).

Variables	Quartiles of HNDI			Quartiles of BSDS		
	Q1	Q4	P-value^*^	Q1	Q4	P-value*
Age (years)	37.8 ± 7.9	40.1 ± 8.1	0.03	39.2 ± 7.3	41.4 ± 8.1	0.027
Male (%)	45.2	53.1	0.064	50.2	46.8	0.071
BMI (Kg/m^2^)	26.3 ± 2.9	26.5 ± 3.0	0.178	26.7 ± 3.1	27.2 ± 3.3	0.128
Smoking, (yes, %)	4.2	2.3	0.147	3.6	3.9	0.698
Physical activity (MET/min/week)	1641.24 ± 785.11	1457.32 ± 622.47	0.134	1472.48 ± 647.11	1413.47 ± 612.84	0.274
Waist-circumference (cm)	98.27 ± 12.30	97.71 ± 12.18	0.327	96.34 ± 12.17	97.07 ± 12.35	0.411
Education (%)			0.012			0.028
Less than a diploma	17.5	15.6		19.2	15.8	
Diploma	38.1	39.1		33.2	38.7	
Bachelor	28.6	20.2		31.8	21.2	
Higher than Bachelor	15.8	45.1		15.8	24.3	

Values are expressed as means [standard deviation (SD)].

*P-values are resulted from the student t-test.

**Table 2** details dietary intake among subjects across BSDS and HNDI quartiles. Compared to the lowest HNDI quartile, the highest quartile showed higher intakes of energy, carbohydrates, proteins, fats, SFA, MUFA, PUFA, cholesterol, fiber, potassium, iron, calcium, magnesium, zinc, vitamins C, E, D, B9, caffeine, and all food groups; sodium intake showed no significant differences. Similarly, the highest BSDS quartile showed higher consumption of energy, carbohydrates, proteins, PUFA, fiber, potassium, iron, calcium, magnesium, zinc, vitamins C, E, D, B9, caffeine, total dairy, legumes, nuts, fish, whole grains, fruits, and vegetables, along with lower red and processed meat intake.

**Table 2:** Dietary intake across the Quartiles of Healthy Nordic Diet Index (HNDI) and Baltic Sea Dietary Score (BSDS).

Variables	Quartiles of HNDI			Quartiles of BSDS		
	Q1	Q4	P-value^*^	Q1	Q4	P-value*
Dietary intake						
Energy (Kcal/d)	1852.84 (512.35)	2465.24 (602.37)	<0.001	1921.47 (563.08)	2391.24 (685.38)	<0.001
Carbohydrate (g/d)	263.41 (83.28)	395.17 (85.64)	<0.001	241.64 (84.90)	334.76 (106.94)	<0.001
Protein (g/d)	59.37 (20.54)	98.57 (31.41)	<0.001	65.24 (25.02)	89.23 (31.96)	<0.001
Fat (g/d)	62.20 (24.18)	88.24 (27.21)	<0.001	75.05 (29.24)	24.18 (25.27)	0.741
SFA (g/d)	22.62 (9.47)	28.07 (10.47)	<0.001	24.56 (10.41)	24.12 (9.20)	0.187
MUFA	21.74 (8.57)	28.31 (10.42)	<0.001	25.54 (10.13)	24.21 (9.78)	0.321
PUFA	13.95 (6.10)	19.64 (9.68)	<0.001	15.38 (8.03)	16.25 (8.15)	0.035
Cholesterol (mg/d)	188.45 (123.85)	295.32 (150.04)	<0.001	224.75 (119.11)	247.27 (137.19)	0.084
Fiber (g/d)	25.41 (18.24)	39.78 (19.66)	<0.001	25.58 (17.75)	39.70 (18.49)	<0.001
Sodium (mg/d)	3985.78 (3087.73)	4199.38 (2745.50)	0.456	4117.10 (382.61)	4124.41 (384.57)	0.478
Potassium (mg/d)	2321.18 (821.96)	4582.14 (1123.89)	<0.001	2741.81 (971.40)	4387.34 (1342.20)	<0.001
Iron (mg/d)	24.88 (9.61)	41.74 (12.61)	<0.001	24.25 (26.60)	38.84 (36.72)	0.001
Calcium (mg/d)	981.01 (375.20)	1267.17 (471.08)	<0.001	960.37 (446.00)	1113.98 (450.24)	<0.001
Magnesium (mg/d)	251.45 (84.71)	428.47 (112.03)	<0.001	273.28 (92.07)	386.18 (134.78)	<0.001
Zinc (mg/d)	9.02 (2.87)	13.57 (3.61)	<0.001	9.71 (3.16)	11.98 (3.38)	<0.001
Vitamin C(mg/d)	91.36 (53.80)	212.44 (87.70	<0.001	97.66 (63.08)	196.60 (97.76)	<0.001
Folate (mcg/d)	399.28 (161.88)	528.76 (190.57)	<0.001	402.20 (160.09)	525.02 (184.67)	<0.001
Vitamin E (mg/d)	8.96 (3.70)	11.62 (4.14)	<0.001	9.66 (4.79)	11.38 (4.96)	0.006
Vitamin D (mcg/d)	1.42 (0.60)	2.31 (0.91)	<0.001	1.37 (1.36)	1.92 (1.48)	0.001
Caffeine (mg/d)	99.48 (69.08)	127.29 (97.83)	0.001	109.24 (74.43)	132.47 (93.25)	0.017
Food groups						
Total dairy (g,;d)	331.62 (241.67)	434.19 (274.58)	<0.001	353.71 (219.32)	417.20 (232.63)	0.065
Legume (g,;d)	8.94 (8.17)	30.97 (24.14)	<0.001	15.22 (19.51)	24.34 (25.89)	<0.001
Nut (g,;d)	5.78 (6.04)	13.16 (14.94)	<0.001	6.85 (8.880	11.84 (12.24)	<0.001
Fish (g,;d)	5.34 (5.44)	16.04 (20.47)	<0.001	6.03 (6.21)	13.65 (12.02)	<0.001
Whole grains (g,;d)	52.34 (69.74)	112.87 (82.40)	<0.001	49.32 (53.97)	115.67 (94.11)	<0.001
Refined grains (g,;d)	362.97 (205.26)	284.64 (175.29)	0.005	281.06 (191.52)	267.94 (164.43)	0.367
Red and Processed meat (g,;d)	25.58 (26.69)	42.78 (34.12)	<0.001	37.38 (32.84)	29.61 (30.44)	0.037
Fruits (g,;d)	234.42 (274.11)	682.12 (395.74)	<0.001	251.67 (231.52)	623.69 (344.31)	<0.001
Vegetables (g,;d)	174.62 (101.60)	384.78 (162.94)	<0.001	204.25 (127.93)	361.68 (162.89)	<0.001

Values are expressed as means [standard deviation (SD)].

*P-values are resulted from the student t-test.

**Table 3** shows odds ratios (ORs) and 95% confidence intervals (CIs) for T2DM, categorized by BSDS and HNDI quartiles. While initial adjusted models (controlling for age and sex) showed no significant association between HNDI and T2DM (OR = 0.99, 95% CI 0.67–1.47; p for trend = 0.870; OR = 0.95, 95% CI 0.64–1.41; p for trend = 0.957), the final model (accounting for confounding variables) linked increased HNDI adherence to a reduced likelihood of T2DM (OR = 0.42; 95% CI 0.18–0.98; p for trend = 0.043). A significant association between lower T2DM likelihood and higher BSDS scores was found in both unadjusted (OR = 0.49, 95% CI 0.31–0.77; p for trend = 0.001) and adjusted (OR = 0.48, 95% CI 0.32–0.89; p for trend = 0.003) models.

**Table 3:** Odds ratio (OR) and 95% confidence interval (CI) for T2DM based on Healthy Nordic Diet Index (HNDI) and Baltic Sea Dietary Score (BSDS).

variable	Quartiles of scores				
	Q1	Q2	Q3	Q4	P for trend
HNDI					
Case/Control	76/113	58/113	49/111	42/113	
Crude model	1.00 (Ref)	0.65 (0.41-1.25)	0.91 (0.60-1.42)	0.93 (0.64-1.31)	0.745
Model 1*	1.00 (Ref)	0.61 (0.42-1.13)	0.78 (0.55-1.27)	0.90 (0.60-1.28)	0.814
Model 2†	1.00 (Ref)	0.62 (0.32-1.48)	0.54 (0.24-1.22)	0.41 (0.22-0.94)	0.039
BSDS					
Case/Control	81/113	62/111	55/113	27/113	
Crude model	1.00 (Ref)	0.89 (0.65-1.48)	0.73 (0.45-1.23)	0.53 (0.34-0.74)	0.001
Model 1*	1.00 (Ref)	0.84 (0.57-1.39)	0.62 (0.40-1.18)	0.45 (0.27-0.71)	0.001
Model 2†	1.00 (Ref)	0.86 (0.51-1.33)	0.61 (0.35-1.12)	0.49 (0.34-0.85)	0.004

Binary logistic regression was used to obtain OR and 95% CI.

*Model 1: adjusted for age and sex. †Model 2: Model 1 + BMI, Waist circumference, hip circumference, physical activity, smoking, education, fiber, and energy intake.

## 4 Discussion

This study investigated the association between the risk of type 2 diabetes and compliance with the Healthy Nordic Diet Index (HNDI) and the Baltic Sea Diet Score (BSDS). Dietary intake was assessed using validated questionnaires and structured interviews, comparing individuals newly diagnosed with type 2 diabetes to healthy controls through a case-control study design. Various contributing variables, including age, sex, and body mass index, were considered using logistic regression in the statistical analysis. The results indicated that higher scores on the Healthy Nordic Diet Index were inversely associated with the risk of diabetes, although the original research did not find a significant correlation between the diet and diabetes risk. Similarly, both before and after adjusting for confounding factors, a strong negative association was identified between higher Baltic Sea Diet Scores and the risk of type 2 diabetes. Further research is necessary to validate these findings, as they suggest that adherence to both dietary patterns may confer protective effects against the development of type 2 diabetes.

Prospective cohort study was conducted by Lacoppidan et al. (2015) within the cohort of Danish Diet, Cancer and Health (n=57,053 aged 50-64 years, mean follow up 15.3 years) who showed that increased adherence to the Healthy Nordic Food Index is associated with significantly reduced risk of T2DM (HR per unit increase 0.94 [95% CI 0.92-0.97] for women and 0.91 [95% CI 0.89-0.93] for men) (16). Likewise, Tertsunen et al. (2021), in a Finnish cohort (n=2,332 men, 42 to 60 years of age, mean follow up 21.9 years), also observed that prospective conformity to a healthy Nordic diet (HR for lowest *vs*. highest quartile: 1.35 [95% CI: 1.03–1.76]) increases T2DM risk (8). Shi et al. (2022) conducted a nested case control study in Sweden (n=421 case: control pairs) to identify T2DM risk metabolites related to HNFI and BSDS. Confirming a reduced T2DM risk (pooled relative risk [RR]: 0.85 [95% CI: 0.77-0.94]) (11), Paraskevi Massara et al., 2022 were able to perform a systematic review and meta–analysis of longitudinal and randomized controlled trials of Nordic dietary patterns (17). A cross-sectional study (n= 339 T2DM patients) was conducted by Daneshzad et al. (2018) wherein their findings revealed better cardiovascular risk factors with higher adherence to modified Nordic diet (18). Kaneria et al. (2014) conducted a meta analysis of three Finnish cross sectional studies (n=4,776–5,180) and showed that higher BSDS adherence improves lipid profiles as well as high sensitivity C reactive protein (hs CRP) (19). In another cross sectional study (n = 4, 776) of Kaneria et al. (2013), BSDS scores were found to correlate with abdominal OB which is the T2DM risk factor (20). Kaneria et al. (2014) lastly describe the BSDS tool and a methodological framework to support the application of the BSDS tool in dietary assessments (14). Together, these studies provide corroboration that meeting Nordic dietary patterns as assessed by HNDI or BSDS is linked with reduced metabolic conduct including decreased T2DM event rate or reduced risk for related factors. In particular, the current study, with a case control design extends this body of evidence by inclusion of a younger group (18 to 60 years) as population rather than exclusively older cohorts, seemingly making its implication more broad. By establishing its methodological rigor with evidence of its use of a comprehensive 168 item food frequency questionnaire (FFQ), adjustment for confounders plus advantages of cohort design over case control design (namely, removing potential recall bias less common in such studies as Lacoppidan et al., (16) where strong causal inference at the cost of extended follow up duration), Lacoppidan et al. achieve their conclusion. Lastly, the findings of the current study are in line with the existing literature and indicate that healthy Nordic dietary patterns can be preventive of T2DM, and further longitudinal investigations on diverse populations are needed to further validate these observations.

A robust body of evidence links nutritional factors to the risk of T2DM, with diet playing a significant role in the pathogenesis and management of the disease (21). Major risk factors include obesity, physical inactivity, genetic predisposition, and suboptimal dietary patterns (22). The Mediterranean diet, which emphasizes the consumption of monounsaturated fats over saturated fats and is characterized by a high intake of fruits, vegetables, whole grains, legumes, nuts, and olive oil, is particularly recognized for its preventive benefits against type 2 diabetes. Adherence to this dietary pattern is significantly inversely correlated with the incidence of T2DM (23), significantly enhances glucose metabolism and reduces markers of oxidative and inflammatory stress (24). Likewise, the Baltic Sea Diet and the Healthy Nordic Food Index have demonstrated positive outcomes by emphasizing the consumption of local and seasonal foods, such as rye, berries, and root vegetables, alongside staples like whole grains and seafood (25). These dietary patterns help to mitigate inflammation and enhance insulin sensitivity, potentially lowering the risk of type 2 diabetes. Both diets emphasize the consumption of foods rich in fiber and bioactive compounds while maintaining a low glycemic load, which may facilitate improved glycemic control and promote metabolic health (26). Consequently, incorporating elements of these diets may prove advantageous for individuals with type 2 diabetes or those at risk, offering a contextually relevant approach to dietary preventive strategies against chronic metabolic disorders (27).

Emerging evidence suggests that adherence to the Baltic Sea Diet and the Healthy Nordic Food Index may significantly decrease the risk of T2DM by enhancing insulin sensitivity and glucose metabolism (28). Both dietary patterns prioritize the consumption of substantial quantities of whole grains, salmon, berries, fruits, and vegetables, which are associated with beneficial hypoglycemic effects (29). High-fiber whole grains, such as rye and barley, integral to the Baltic Sea Diet, play a crucial role in maintaining stable blood glucose levels and reducing carbohydrate absorption, thereby alleviating insulin resistance (30). Likewise, the Healthy Nordic Food Index promotes the intake of low-glycemic index and high-fiber foods, including legumes and root vegetables, which further support glucose regulation. Additionally, a key component of both diets, omega-3 fatty acids derived from fish, significantly reduce inflammation—a critical factor in the pathophysiology of T2DM—and enhance cellular insulin action (31). The antioxidant and anti-inflammatory properties of berries contribute to the reduction of oxidative stress, thereby enhancing insulin sensitivity (32). Furthermore, both dietary patterns ensure a balanced intake of macronutrients, including beneficial fats derived from nuts and seeds, which support optimal lipid profiles and protect against insulin resistance. Collectively, these dietary elements from the Healthy Nordic Food Index and the Baltic Sea Diet facilitate improvements in lipid and glucose metabolism, mitigate inflammation, and enhance overall metabolic health, thereby reinforcing their protective effects against type 2 diabetes (33).

Certain dietary components of the Healthy Nordic Food Index and the Baltic Sea Diet significantly diminish the risk of T2DM. Staples of these diets, including fish, berries, fruits, vegetables, and whole grains, each contribute to the reduction of T2DM risk through distinct mechanisms (34). Whole grains, such as rye, barley, and oats, are particularly notable for their high soluble fiber content and magnesium levels, which facilitate insulin action and glucose metabolism (35). Soluble fiber effectively lowers blood glucose levels by decelerating carbohydrate absorption, enhancing glycemic control, and improving insulin sensitivity. Additionally, fatty fish, including mackerel and salmon, are abundant in omega-3 fatty acids, which enhance insulin signaling, reduce inflammation, and alleviate insulin resistance and dyslipidemia associated with T2DM (36). Berries and fruits, characterized by their high antioxidant content and low glycemic index, play a significant role in mitigating oxidative stress and preventing postprandial blood glucose fluctuations. Furthermore, whole grains such as rye, barley, and oats are not only abundant in soluble fiber but also contain magnesium, which facilitates insulin action and glucose metabolism (35). Soluble fiber contributes to the regulation of blood sugar levels by decelerating sugar absorption, thereby enhancing glycemic control and improving insulin sensitivity (37).

Lower adherence to this dietary pattern is associated with an increased risk of diabetes, as well as elevated plasma glucose and insulin levels. This finding aligns with the research conducted by Tertsunen et al, which examined the relationship between adherence to a healthy Nordic diet and the risk of type 2 diabetes in middle-aged and older men (8). The utilization of food records to assess dietary intake highlights the significance of thorough dietary evaluations in understanding diabetes risk. Additionally, the extended follow-up period of the study underscores the necessity for sustained dietary adherence, indicating that dietary strategies emphasizing nutrient diversity may be pivotal in the prevention and management of diabetes.

Even after accounting for other factors, Lacoppidan et al.’s study confirmed a strong association between adherence to a healthy Nordic diet and a lower risk of type 2 diabetes in Danish men and women. This confirms a link between local diets and disease prevention and highlights the potential of diverse healthy dietary patterns, beyond the Mediterranean diet, in preventing type 2 diabetes (16).

Lin Shi et al. investigated the link between dietary indices (HNFI and BSDS) and type 2 diabetes mellitus (T2DM) risk. While individuals who later developed T2DM showed more baseline risk factors, adherence levels to the indices didn’t significantly alter risk, despite increased food consumption with higher adherence. While certain metabolites linked to the dietary indices (such as gamma-tocopherol and docosahexaenoic acid) initially appeared to reduce T2DM risk, this association diminished after accounting for body mass index and cholesterol. This finding indicates that healthy eating habits alone may not completely determine T2DM risk, emphasizing the intricate relationship between dietary components and T2DM development (11).

Kanerva et al. developed the Balanced Dietary Score (BSDS) to assess the Nordic dietary pattern’s impact on health. Using data from the National FINRISK 2007 Study and a validated food frequency questionnaire, the BSDS showed that higher adherence correlates with increased carbohydrate intake, reduced alcohol and saturated fat intake, and increased fiber, iron, and other essential nutrients, even after adjustments. This indicates the BSDS is a useful tool for evaluating diet-health relationships in Nordic public health surveys (14).

Daneshzad et al.’s cross-sectional study of 339 type 2 diabetes patients examined the relationship between a modified Nordic diet and cardiovascular risk factors. A potential benefit of a Nordic diet for cardiovascular health and obesity reduction in type 2 diabetes is suggested by findings showing that higher diet adherence, influenced by socioeconomic status, was associated with lower BMI, lower systolic blood pressure, lower LDL cholesterol, and lower aspartate aminotransferase levels. However, further research is needed (18).

This case-control study investigated the association between the Baltic Sea Diet Score (BSDS) and Healthy Nordic Diet Index (HNDI) and type diabetes mellitus (T2DM) risk in year olds diagnosed with T2DM within the past six months and age-matched healthy controls. Dietary intake was assessed using a -item semi-quantitative food frequency questionnaire. Higher BSDS and HNDI quartiles were associated with higher intakes of various nutrients and food groups considered beneficial, and lower intakes of less healthy options (e.g., red and processed meats). After adjusting for confounders, higher HNDI was associated with a reduced likelihood of T2DM, and higher BSDS was also significantly associated with a lower risk of T2DM.

The comprehensive dietary evaluation executed through this case-control study the use of questionnaires, interviews, and anthropometric measurements is one in all its principal strengths. It allows an in-intensity evaluation of the nutritional practices of individuals with type 2 diabetes and wholesome controls. However, the use of self-said information introduces take into account bias. Selection bias may additionally have resulted from the examiner’s rigorous exclusion criteria, or even as confounding variables were accounted for, a few may still exist. The assessment of statistical power and generalizability is hampered by the lack of sample length records, and the inability to establish precise timing regarding dietary intake and onset of disease restricts causal findings.

## 5 Conclusions

Our research findings have clearly indicated that strict adherence to a healthy Nordic diet significantly lowers the risk of developing type 2 diabetes mellitus (T2DM). This demonstrates that the highly nutritious and beneficial components inherent in the Nordic diet play a crucial role in mitigating the risk associated with T2DM. Consequently, maintaining a strong commitment to a healthy Nordic dietary pattern may also prove effective in decreasing the likelihood of developing various risk factors associated with T2DM. To further validate our findings, additional longitudinal studies involving a diverse range of populations are necessary and warranted.

## Data Availability

The raw data supporting the conclusions of this article will be made available by the authors, without undue reservation.
